# Linear Regression in Genetic Association Studies

**DOI:** 10.1371/journal.pone.0056976

**Published:** 2013-02-21

**Authors:** Petra Bůžková

**Affiliations:** Department of Biostatistics, University of Washington, Seattle, Washington, United States of America; Yale School of Public Health, United States of America

## Abstract

In genomic research phenotype transformations are commonly used as a straightforward way to reach normality of the model outcome. Many researchers still believe it to be necessary for proper inference. Using regression simulations, we show that phenotype transformations are typically not needed and, when used in phenotype with heteroscedasticity, result in inflated Type I error rates. We further explain that important is to address a combination of rare variant genotypes and heteroscedasticity. Incorrectly estimated parameter variability or incorrect choice of the distribution of the underlying test statistic provide spurious detection of associations. We conclude that it is a combination of heteroscedasticity, minor allele frequency, sample size, and to a much lesser extent the error distribution, that matter for proper statistical inference.

## Introduction

Phenotype transformations are still very popular in genomic research when drawing inference about genotype-phenotype associations. The two most widely used transformations are natural logarithm and rank-based inverse-normal transformation (INT). Their popularity is underlined by them being easily obtained; logarithm has an automated function in all statistical software packages and INT in many, for example, in SAS and SPSS. Often the only reason to transform a phenotype is “to improve normality”. Many researchers still believe that linear regression is valid only for normally distributed model residuals or even model outcomes. We give a few examples: “the Von Willebrand factor was natural log-transformed to improve normality” [1]; “Weight and body mass index scores were transformed by the natural logarithm to normalise the distribution” [2]; “Natural logarithm transformations were used to improve normality of the distributions for HDL and LDL cholesterol, TG, and BMI” [3]. Sometimes phenotypes are transformed without providing any reason at all [4]. Phenotype transformations alter the regression model and the interpretation of the parameter estimates. There is no direct way how to translate the parameter estimates back into difference in the mean of the phenotype, but attempts to do so are many, e.g. [5]. Nevertheless, in the absence of believes about biological plausibility and in the absence of a detailed scientific question to guide the model choice, a phenotype transformation could be chosen for statistical convenience, if merit.

Linear regression is frequently presented as an optimal method in the context of normally distributed model residuals. But it was discovered as a semiparametric method based on least squares. The fact that residual normality is not necessary for validity of linear regression in sufficiently large samples is well understood in the statistical literature but it is still not widely known and accepted in genetic epidemiology. The sample size in genetic epidemiology studies is typically in thousands, using established cohorts. If study data are stratified for analyses by race/ethnicity and sometimes additionally by an environmental exposure, the sample size is still at least in several hundreds. The behavior of t-tests with extremely non-normal medical cost data suggests that major limitation is not distributional but whether detecting and estimating a difference in the mean of the outcome answers the scientific question at hand [6]. Simulations for two-group comparisons showed lack of justification for INTs in most situations [7].

In contrast to two-group comparison tests, there has been little empirical research into the behavior of linear regression. In this paper we first show that in linear regression in the genetic association studies framework transformations are typically not needed to improve adherence of the Type I error rate to the nominal level. Further, we focus on a regression feature unique to genetic data. Genotype, the major covariate of interest characterized by minor allele frequency (MAF), is prone to skewness. Many genetic studies include single nucleotide polymorphisms (SNPs) with low MAF, often as low as 0.05 or 0.01, known as rare variants. New arrays are being developed specifically for fine mapping, targeted at SNPs with lower MAF. For instance, Metabochip is a custom SNP array based on the Illumina iSelect platform and was developed for replication and fine mapping of susceptibility variants associated with several metabolic and cardiovascular traits. The MAF filters there may be set as low as 0.001, resulting in genotype skewness of 22. With rare variants addressing of heteroscedasticity becomes crucial. In this paper we discuss the choice of standard errors (SEs) to construct a test statistic and the choice of the distribution of the test statistic under the null hypothesis that improve adherence of the Type I error rate to the nominal level.

We note that techniques involving collapsing or summing rare variants, often referred to as pooling or burden tests [8], [9], are another route of addressing the situation with rare variants. The lack of robustness of such methods to neutral and protective variants in comparison to single variant test statistics has recently been noted [10].

To illustrate the situation, denote 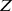 a positive continuous phenotype, such as body mass index (BMI) or plasma lipid concentrations. We define three model outcomes 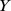: The untransformed phenotype 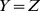; The natural logarithm of the phenotype 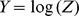; A rank-based INT of the phenotype 
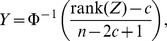
 with 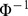 denoting the standard normal quantile function and 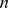 the sample size. We take 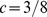 [7], [11]. The typical linear regression model in a genetic association study is.
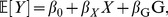
(1)where 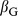 is the parameter of interest quantifying the association between a genotype G and the mean of an outcome 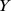. Further, 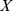 is a small set of 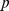 covariates, such as age and gender. Denote 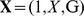 and 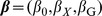. As long as the model for the mean of the outcome (1) is correct, the ordinary least squares estimator 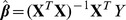 is an unbiased estimator of 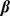. It is a weighted average of the model outcome 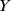 with weights that dependent on the covariate set 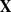.

We consider a test for no association between the genotype G and the mean of the model outcome 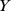, i.e., 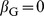 in model (1). The natural test statistic is the standardized score obtained by dividing the parameter estimate 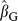 by its estimated standard error. For unbiased estimation of the standard error we need either assumptions on variance of the outcome or to use an empirical estimator. The model-based estimator is based on the classical assumption of constant outcome variance and is the one being typically used. The other is robust to that assumption, empirically estimating the variability of the outcome [12], [13]. This method is most widely used in the context of the generalized estimating equations [14]. It is appropriate to use when heteroscedasticity is present, when variability of the outcome given covariates is a function of the covariates rather than constant. This is specially true with skewed covariates, such as SNPs with low MAF. On the other hand this robust standard error estimator may perform suboptimal for very small effective sample sizes (small sample size, skewed covariates), being inefficient [15]–[17]. For larger effective sample sizes, however, regardless of the actual outcome variability, this robust estimator is always valid. Heteroscedasticity does not cause bias in the parameter estimator itself. It can only cause the estimators of the variability of the parameter estimate to be inconsistent. Confidence intervals and tests' p-values may be invalid. Model-based standard errors as compared to robust standard errors were recently discussed in the context of gene-environment interaction in GWAS [18]. Population substructure was suggested by elevated median interaction test statistic when model-based standard errors were used. The inflation was alleviated when robust standard errors were used.

Testing in a regression model framework requires specification of the distribution of the test statistic under the null-hypothesis. The asymptotic distribution of the test statistic is standard normal, and another is a 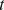-distribution with 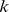 degrees of freedom. Using a 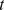-distribution has impact for smaller 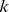 because it is approaching the standard normal distribution with increasing 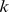. Having heavier tails, it will provide wider confidence intervals and larger 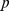-values. When using the model-based standard errors, the degrees of freedom are 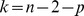. As an analogy to the Welch-Satterthwite approximation of degrees of freedom in a t-test under heterogeneous variance [19], a Lipsitz formula provides an approximation of number of degrees of freedom in linear regression when using the robust standard errors [20]. The computation of the degrees of freedom involves estimation of the variability of the variance estimator for each parameter estimate and is different for different parameter estimates within a single regression model. Unlike the Welch-Satterthwite formula for the t-test, we are not aware of any statistical software package making the Lipsitz formula for linear regression readily available. We approximate the effective degrees of freedom 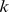 with 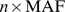.

An improper choice of the distribution of the test statistic under the null-hypothesis has consequences for inference validity: if distributional quantiles are too small, as could with normal quantiles or overestimated effective degrees of freedom of a 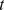-distribution, decisions may be uncoservative with too low p-values, resulting in spurious detection of associations; if distributional quantiles are too large, as could with underestimated effective degrees of freedom of a 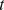-distribution, decisions may be conservative with too large p-values, failing to discover the associations.

In moderate to large sample sizes, there exists a third option for specification of the distribution of the test statistic under the null-hypothesis; Parametric bootstrap [21, 4.2.3] can provide a good approximation to the distribution of the test statistic under sampling from the true null-hypothesis model through the distribution of the test statistic under sampling from the fitted null-hypothesis model. However, similarly to any resampling approach, this option typically requires additional coding and may be unfeasible with larger number of models. We do not consider it in our paper.

## Results

Using simulation, we explore tests of association between a genotype and a phenotype in linear regression in samples of unrelated individuals. For a range of sample sizes and MAFs we consider two types of error distributions with heavy tails - Weibull and 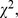 and, for reference, normal distribution. We simulate non-heteroscedastic and heteroscedastic data sets. We evaluate the impact of phenotype transformations and compare three statistical approaches for p-value computation. We also discuss power. We demonstrate the methods with an actual genetic association study data.

### No Heteroscedasticity

**Figure 1:**
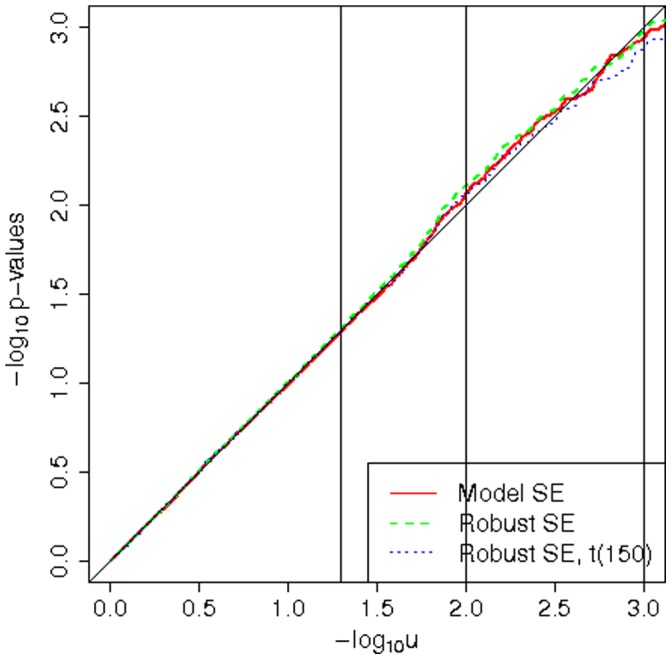
Typical scenarios with no heteroscedasticity. A sample QQ plot for sample size of 500 and MAF 0.3.

Figure 1 for sample size of 500 and MAF 0.3 shows the typical results for any of the error distribution. Phenotype transformations were not needed to improve the Type I error rate adherence to the nominal level. All three approaches, that is model-based SEs combined with normal quantiles, robust SEs with normal quantiles and robust SEs with t-quantiles with 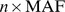 approximate effective degrees of freedom, performed similarly well showing adherence of the Type I error rate to the nominal level. Tables 1 and 2 summarize the type I error rates across MAF and sample size for 5% and 0.1% significance levels.

**Table 1:** Table of type I error rates for normally distributed errors with no heteroscedasticity.

		no transformation						transformation					
MAF	n	α = 0.05			α = 0.001			α = 0.05			α = 0.001		
		M1	M2	M3	M1	M2	M3	M1	M2	M3	M1	M2	M3
0.01	200	0.054	0.246	0.085	0.0013	0.1240	0.0000	0.054	0.246	0.086	0.0013	0.1234	0.0000
	500	0.052	0.100	0.044	0.0015	0.0171	0.0014	0.051	0.100	0.045	0.0012	0.0165	0.0014
	1000	0.046	0.073	0.043	0.0008	0.0047	0.0003	0.046	0.073	0.043	0.0008	0.0044	0.0003
	2000	0.050	0.062	0.050	0.0010	0.0022	0.0005	0.050	0.061	0.049	0.0010	0.0022	0.0003
	5000	0.046	0.052	0.046	0.0014	0.0013	0.0007	0.047	0.051	0.046	0.0015	0.0013	0.0007
0.05	200	0.049	0.070	0.042	0.0010	0.0045	0.0002	0.050	0.070	0.043	0.0011	0.0042	0.0002
	500	0.052	0.064	0.051	0.0010	0.0028	0.0008	0.052	0.064	0.050	0.0011	0.0027	0.0007
	1000	0.051	0.053	0.047	0.0011	0.0020	0.0009	0.050	0.053	0.047	0.0012	0.0019	0.0010
	2000	0.050	0.053	0.050	0.0011	0.0012	0.0009	0.050	0.053	0.050	0.0011	0.0012	0.0009
	5000	0.054	0.055	0.054	0.0012	0.0012	0.0012	0.054	0.055	0.054	0.0013	0.0012	0.0012
0.1	200	0.049	0.061	0.045	0.0008	0.0020	0.0002	0.049	0.061	0.044	0.0008	0.0020	0.0002
	500	0.048	0.052	0.046	0.0016	0.0020	0.0014	0.048	0.052	0.047	0.0016	0.0020	0.0013
	1000	0.051	0.054	0.051	0.0013	0.0017	0.0012	0.051	0.053	0.051	0.0012	0.0017	0.0011
	2000	0.051	0.052	0.051	0.0013	0.0016	0.0015	0.051	0.052	0.051	0.0014	0.0016	0.0015
	5000	0.048	0.047	0.046	0.0007	0.0008	0.0006	0.047	0.047	0.047	0.0007	0.0008	0.0007
0.3	200	0.051	0.055	0.050	0.0008	0.0010	0.0006	0.051	0.054	0.050	0.0008	0.0010	0.0006
	500	0.049	0.051	0.049	0.0008	0.0010	0.0006	0.049	0.051	0.049	0.0009	0.0010	0.0006
	1000	0.050	0.052	0.052	0.0011	0.0010	0.0010	0.051	0.053	0.052	0.0011	0.0010	0.0010
	2000	0.050	0.051	0.050	0.0006	0.0008	0.0007	0.049	0.051	0.050	0.0006	0.0007	0.0006
	5000	0.051	0.051	0.051	0.0014	0.0014	0.0014	0.051	0.052	0.051	0.0013	0.0014	0.0013
0.5	200	0.055	0.060	0.056	0.0018	0.0027	0.0015	0.054	0.060	0.056	0.0017	0.0025	0.0015
	500	0.051	0.052	0.052	0.0008	0.0011	0.0009	0.050	0.052	0.052	0.0007	0.0011	0.0010
	1000	0.051	0.053	0.052	0.0012	0.0012	0.0012	0.051	0.053	0.052	0.0012	0.0012	0.0012
	2000	0.052	0.053	0.052	0.0010	0.0010	0.0010	0.052	0.053	0.052	0.0010	0.0010	0.0010
	5000	0.053	0.053	0.053	0.0016	0.0016	0.0016	0.054	0.054	0.054	0.0016	0.0016	0.0016

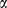
 is significance level. M1 uses model-based SEs combined with normal quantiles. M2 uses robust SE with normal quantiles. M3 uses robust SEs with t-quantiles with 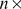 MAF degrees of freedom.

**Table 2:** Table of type I error rates for Weibull errors with no heteroscedasticity.

		no transformation						transformation					
MAF	n	α = 0.05			α = 0.001			α = 0.05			α = 0.001		
		M1	M2	M3	M1	M2	M3	M1	M2	M3	M1	M2	M3
0.01	200	0.045	0.269	0.104	0.0036	0.1489	0.0007	0.046	0.258	0.097	0.0016	0.1377	0.0001
	500	0.048	0.117	0.058	0.0019	0.0298	0.0024	0.051	0.105	0.047	0.0012	0.0227	0.0017
	1000	0.050	0.084	0.055	0.0012	0.0118	0.0021	0.052	0.075	0.049	0.0011	0.0072	0.0012
	2000	0.052	0.069	0.056	0.0009	0.0054	0.0020	0.052	0.063	0.050	0.0007	0.0043	0.0012
	5000	0.051	0.055	0.050	0.0013	0.0030	0.0012	0.049	0.054	0.049	0.0014	0.0019	0.0008
0.05	200	0.050	0.081	0.051	0.0017	0.0083	0.0011	0.050	0.076	0.045	0.0013	0.0062	0.0007
	500	0.051	0.063	0.051	0.0012	0.0031	0.0011	0.051	0.061	0.049	0.0010	0.0022	0.0009
	1000	0.054	0.058	0.054	0.0008	0.0029	0.0015	0.051	0.056	0.050	0.0009	0.0024	0.0014
	2000	0.051	0.054	0.051	0.0009	0.0021	0.0016	0.050	0.052	0.050	0.0008	0.0013	0.0009
	5000	0.049	0.051	0.050	0.0017	0.0017	0.0016	0.049	0.050	0.049	0.0013	0.0017	0.0015
0.1	200	0.050	0.063	0.049	0.0011	0.0035	0.0011	0.052	0.062	0.047	0.0009	0.0026	0.0005
	500	0.050	0.056	0.051	0.0008	0.0010	0.0004	0.051	0.056	0.051	0.0004	0.0011	0.0006
	1000	0.049	0.052	0.049	0.0012	0.0016	0.0014	0.048	0.051	0.049	0.0014	0.0017	0.0015
	2000	0.051	0.053	0.051	0.0004	0.0008	0.0006	0.051	0.053	0.051	0.0006	0.0007	0.0006
	5000	0.053	0.055	0.054	0.0013	0.0016	0.0016	0.053	0.052	0.052	0.0015	0.0014	0.0014
0.3	200	0.051	0.057	0.053	0.0011	0.0020	0.0009	0.049	0.054	0.050	0.0012	0.0018	0.0008
	500	0.050	0.052	0.051	0.0016	0.0017	0.0015	0.050	0.053	0.050	0.0016	0.0020	0.0017
	1000	0.050	0.050	0.049	0.0016	0.0020	0.0019	0.049	0.051	0.050	0.0014	0.0014	0.0013
	2000	0.050	0.052	0.051	0.0012	0.0015	0.0014	0.049	0.051	0.050	0.0012	0.0014	0.0014
	5000	0.050	0.050	0.050	0.0006	0.0006	0.0006	0.052	0.052	0.052	0.0007	0.0006	0.0006
0.5	200	0.053	0.057	0.054	0.0013	0.0018	0.0014	0.052	0.057	0.053	0.0013	0.0017	0.0011
	500	0.049	0.052	0.051	0.0010	0.0011	0.0008	0.048	0.051	0.050	0.0008	0.0009	0.0008
	1000	0.048	0.049	0.048	0.0015	0.0014	0.0013	0.051	0.051	0.050	0.0009	0.0009	0.0008
	2000	0.051	0.052	0.051	0.0009	0.0011	0.0011	0.051	0.051	0.051	0.0008	0.0008	0.0008
	5000	0.051	0.053	0.053	0.0014	0.0014	0.0014	0.054	0.054	0.054	0.0011	0.0010	0.0010

**Figure 2:**
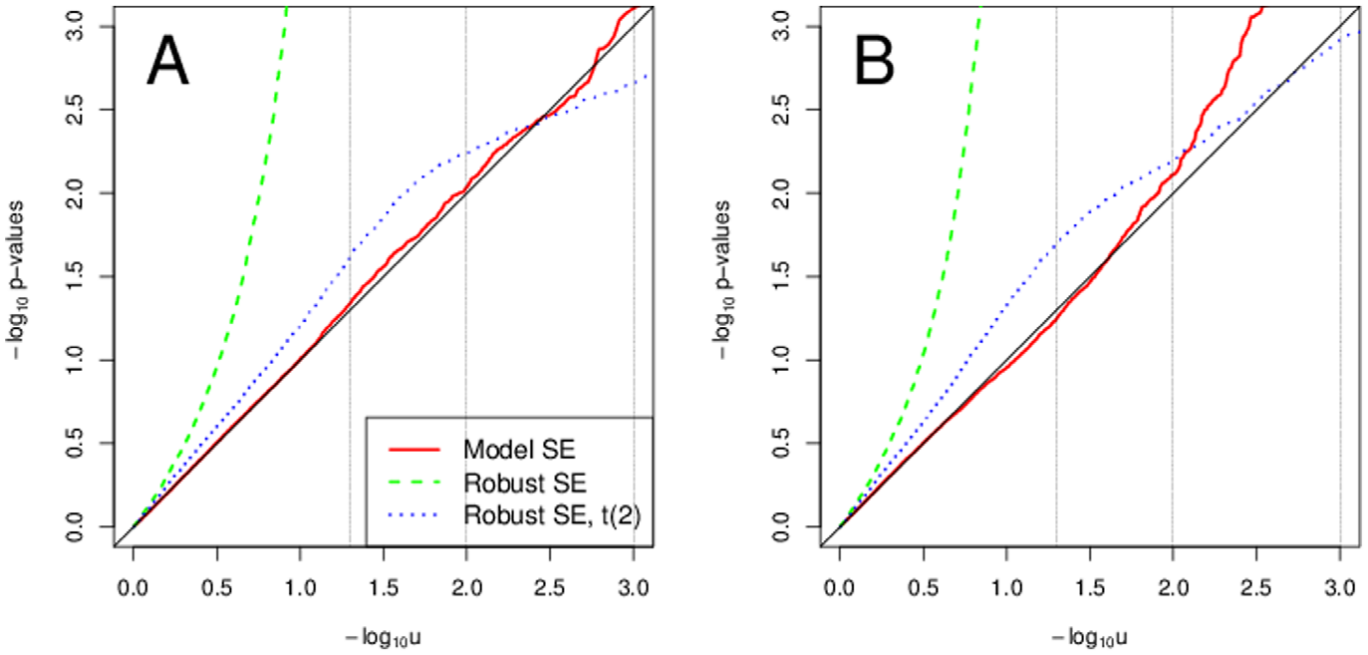
Extreme scenarios with no heteroscedasticity. Sample QQ plots for sample size of 200 and MAF 0.01. In panel A normal errors are used. In panel B 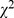 or Weibull errors are used.

We note that for a very small sample size of 200 and rare variants with MAF 0.01 we saw too high Type I error rates when using the robust SEs with normal quantiles, see Figure 2. Using the robust SEs with t-quantiles with degrees of freedom of 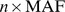 brought the Type I error rates closer to the nominal values. The effective number of degrees of freedom is very low at 2. For Weibull and 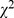 distributed errors the Type I error rates were too large for low significance levels (0.01 and smaller) also when using the model-based SEs. It indicates that in this extreme scenario the asymptotic properties of the test statistic are not in place yet. At the 5% significance level they are, however, still correct. Phenotype transformations improved the Type I error adherence when using the model-based SEs, but the improvement was slight. When using the robust SEs, there was no improvement with transformations, using any test statistic distribution.

### Heteroscedasticity

**Figure 3:**
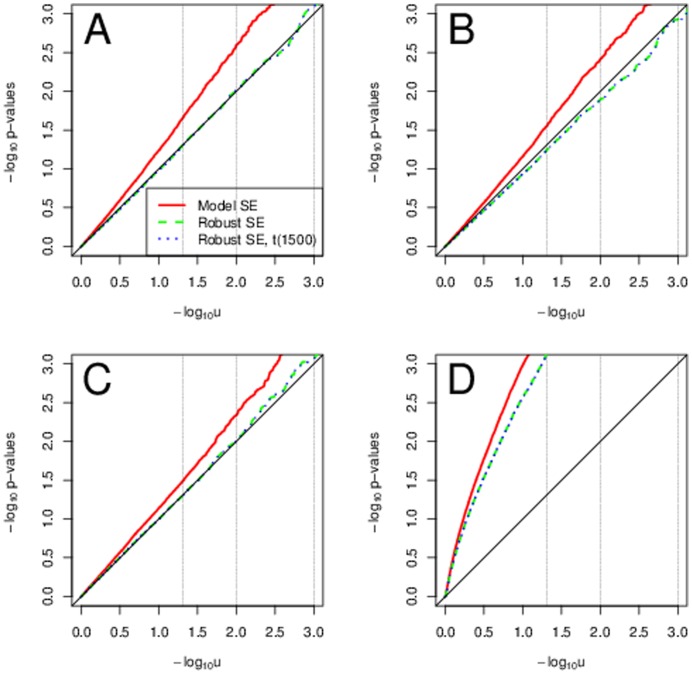
Typical scenarios with heteroscedasticity and some skewness in genotype. Sample QQ plots for sample size of 5000 and MAF 0.3. Panel A uses normal errors and no transformations. Panel B uses normal errors and transformations. Panel C uses 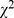 or Weibull errors and no transformations. Panel D uses 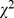 or Weibull errors and transformations.

**Figure 4:**
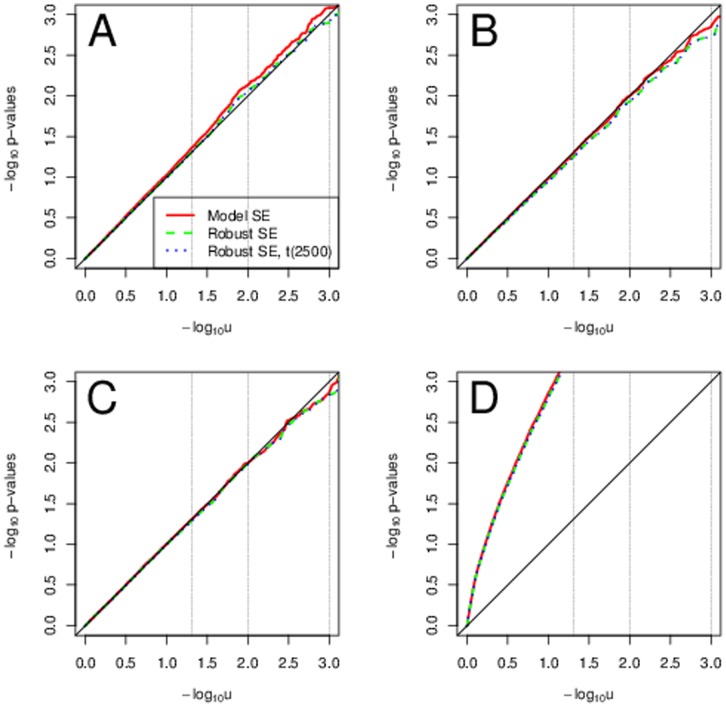
Typical scenarios with heteroscedasticity and no skewness in genotype. Sample QQ plots for sample size of 5000 and MAF 0.5. Panel A uses normal errors and no transformations. Panel B uses normal errors and transformations. Panel C uses 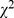 or Weibull errors and no transformations. Panel D uses 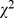 or Weibull errors and transformations.

With heteroscedastic data and some skewness in the genotype, the model-based SEs are not valid estimates of the variability of the estimated parameter and thus using them provides invalid 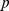-values. Regardless of the sample size we saw too small 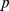-values, i.e, too large Type I error rates. See Figure 3 for sample size of 5000 and MAF 0.3. Transformations did not correct the Type I error rates when using model-based SEs. On the contrary, they seem to increase them even further, specially with the heavy tailed errors with Weibull and 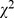 distribution. The 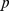-values based on the model-based SEs were improving with increasing MAFs, i.e., with decreasing genotype skewness. With genotype skewness of 0 for MAF 0.5, using model-based SE provided approximately valid inference, see Figure 4. Even in this scenario, with Weibull and 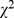 error distribution the transformations provided utterly invalid Type I error rates. With heteroscedasticity in phenotype, using robust standard errors is a valid approach. Yet again, with transformations and Weibull and 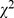 error distribution the Type I errors substantially exceeded their expectation. Tables 3 and 4 summarize the type I error rates across MAF and sample size for 5% and 0.1% significance levels.

**Table 3:** Table of type I error rates for normally distributed errors with heteroscedasticity.

		notransformation						transformation					
MAF	N	α = 0.05			α = 0.001			α = 0.05			α = 0.001		
		M1	M2	M3	M1	M2	M3	M1	M2	M3	M1	M2	M3
0.01	200	0.189	0.268	0.108	0.0282	0.1480	0.0021	0.189	0.268	0.108	0.0283	0.1473	0.0022
	500	0.187	0.104	0.046	0.0264	0.0179	0.0014	0.187	0.104	0.046	0.0255	0.0180	0.0014
	1000	0.184	0.074	0.046	0.0301	0.0051	0.0004	0.185	0.074	0.046	0.0304	0.0052	0.0004
	2000	0.182	0.058	0.044	0.0260	0.0027	0.0003	0.181	0.058	0.044	0.0259	0.0026	0.0003
	5000	0.185	0.056	0.049	0.0286	0.0014	0.0009	0.185	0.057	0.051	0.0280	0.0015	0.0010
0.05	200	0.170	0.077	0.047	0.0219	0.0069	0.0004	0.170	0.077	0.047	0.0217	0.0070	0.0004
	500	0.173	0.061	0.051	0.0216	0.0022	0.0007	0.173	0.062	0.051	0.0215	0.0023	0.0007
	1000	0.163	0.053	0.046	0.0214	0.0020	0.0010	0.164	0.052	0.048	0.0221	0.0021	0.0009
	2000	0.171	0.054	0.050	0.0220	0.0015	0.0012	0.172	0.053	0.050	0.0231	0.0014	0.0012
	5000	0.174	0.054	0.053	0.0210	0.0008	0.0007	0.176	0.055	0.054	0.0222	0.0011	0.0009
0.1	200	0.157	0.069	0.054	0.0176	0.0027	0.0004	0.157	0.070	0.054	0.0177	0.0026	0.0004
	500	0.158	0.061	0.054	0.0179	0.0016	0.0007	0.159	0.060	0.055	0.0185	0.0015	0.0007
	1000	0.161	0.058	0.056	0.0201	0.0018	0.0016	0.162	0.058	0.056	0.0203	0.0020	0.0015
	2000	0.146	0.050	0.048	0.0153	0.0011	0.0009	0.147	0.049	0.048	0.0156	0.0011	0.0010
	5000	0.155	0.053	0.052	0.0176	0.0011	0.0010	0.159	0.057	0.056	0.0181	0.0016	0.0014
0.3	200	0.098	0.059	0.054	0.0055	0.0027	0.0013	0.098	0.060	0.056	0.0057	0.0026	0.0013
	500	0.094	0.053	0.051	0.0052	0.0012	0.0011	0.095	0.054	0.052	0.0061	0.0014	0.0009
	1000	0.094	0.052	0.051	0.0051	0.0010	0.0009	0.093	0.052	0.051	0.0048	0.0009	0.0008
	2000	0.092	0.052	0.051	0.0061	0.0015	0.0015	0.095	0.053	0.053	0.0061	0.0015	0.0015
	5000	0.090	0.050	0.049	0.0049	0.0013	0.0013	0.098	0.052	0.052	0.0059	0.0013	0.0012
0.5	200	0.059	0.058	0.055	0.0018	0.0016	0.0012	0.060	0.059	0.057	0.0020	0.0016	0.0011
	500	0.059	0.053	0.052	0.0017	0.0015	0.0014	0.059	0.053	0.051	0.0022	0.0017	0.0014
	1000	0.058	0.053	0.052	0.0015	0.0013	0.0012	0.059	0.054	0.054	0.0017	0.0013	0.0013
	2000	0.055	0.051	0.051	0.0011	0.0007	0.0007	0.059	0.053	0.053	0.0009	0.0008	0.0008
	5000	0.056	0.050	0.050	0.0013	0.0008	0.0008	0.062	0.056	0.056	0.0022	0.0017	0.0017

**Table 4:** Table of type I error rates for Weibull errors with heteroscedasticity.

		notransformation						Transformation					
MAF	n	α = 0.05			α = 0.001			α = 0.05			α = 0.001		
		M1	M2	M3	M1	M2	M3	M1	M2	M3	M1	M2	M3
0.01	200	0.106	0.269	0.115	0.0095	0.1588	0.0018	0.119	0.261	0.107	0.0065	0.1471	0.0008
	500	0.113	0.120	0.063	0.0101	0.0329	0.0026	0.118	0.116	0.057	0.0090	0.0256	0.0020
	1000	0.110	0.083	0.053	0.0098	0.0103	0.0024	0.114	0.083	0.052	0.0087	0.0081	0.0012
	2000	0.114	0.065	0.051	0.0093	0.0060	0.0022	0.122	0.071	0.056	0.0091	0.0056	0.0017
	5000	0.110	0.058	0.052	0.0087	0.0025	0.0015	0.138	0.075	0.068	0.0142	0.0046	0.0026
0.05	200	0.106	0.083	0.055	0.0080	0.0109	0.0013	0.112	0.086	0.055	0.0066	0.0089	0.0007
	500	0.111	0.064	0.051	0.0071	0.0044	0.0018	0.122	0.074	0.060	0.0088	0.0048	0.0019
	1000	0.110	0.057	0.053	0.0070	0.0028	0.0016	0.136	0.079	0.071	0.0118	0.0045	0.0031
	2000	0.109	0.055	0.052	0.0069	0.0019	0.0011	0.160	0.096	0.093	0.0163	0.0043	0.0031
	5000	0.111	0.053	0.052	0.0084	0.0022	0.0021	0.227	0.140	0.137	0.0293	0.0078	0.0074
0.1	200	0.097	0.068	0.052	0.0066	0.0054	0.0019	0.108	0.075	0.059	0.0069	0.0056	0.0019
	500	0.096	0.058	0.052	0.0055	0.0024	0.0019	0.122	0.075	0.068	0.0086	0.0042	0.0030
	1000	0.100	0.057	0.053	0.0061	0.0018	0.0015	0.146	0.089	0.085	0.0113	0.0053	0.0038
	2000	0.100	0.054	0.053	0.0067	0.0016	0.0014	0.184	0.113	0.111	0.0215	0.0080	0.0068
	5000	0.098	0.053	0.053	0.0073	0.0015	0.0015	0.315	0.217	0.216	0.0539	0.0209	0.0198
0.3	200	0.076	0.062	0.057	0.0029	0.0029	0.0017	0.094	0.081	0.074	0.0048	0.0045	0.0027
	500	0.071	0.053	0.051	0.0024	0.0017	0.0012	0.118	0.095	0.092	0.0087	0.0053	0.0043
	1000	0.072	0.051	0.050	0.0019	0.0010	0.0009	0.172	0.138	0.136	0.0130	0.0079	0.0071
	2000	0.075	0.051	0.051	0.0029	0.0011	0.0010	0.264	0.214	0.213	0.0342	0.0183	0.0177
	5000	0.072	0.049	0.049	0.0032	0.0015	0.0015	0.513	0.450	0.449	0.1234	0.0741	0.0735
0.5	200	0.056	0.057	0.055	0.0016	0.0023	0.0019	0.072	0.076	0.073	0.0031	0.0039	0.0034
	500	0.054	0.052	0.051	0.0016	0.0017	0.0017	0.108	0.107	0.106	0.0053	0.0062	0.0051
	1000	0.053	0.050	0.050	0.0013	0.0012	0.0012	0.157	0.154	0.153	0.0119	0.0114	0.0113
	2000	0.053	0.052	0.052	0.0010	0.0010	0.0010	0.268	0.262	0.262	0.0277	0.0258	0.0254
	5000	0.050	0.048	0.048	0.0008	0.0008	0.0008	0.551	0.544	0.544	0.1227	0.1156	0.1145

**Figure 5:**
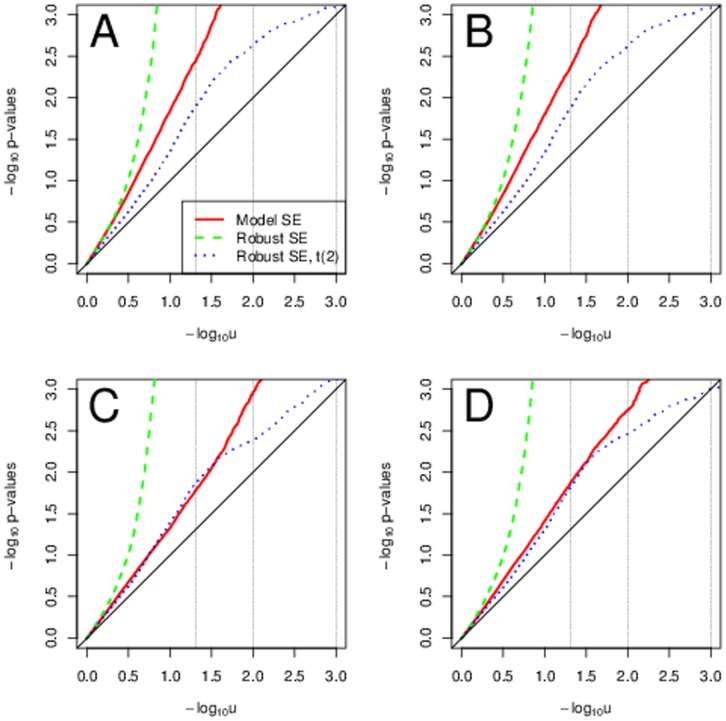
Typical scenarios with heteroscedasticity and rare genetic variants. Sample QQ plots for sample size of 200 and MAF 0.01. Panel A uses normal errors and no transformations. Panel B uses normal errors and transformations. Panel C uses 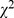 or Weibull errors and no transformations. Panel D uses 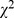 or Weibull errors and transformations.

Using robust standard errors is a valid approach, though with a very small sample size of 200 and rare variants with MAF 0.01 this empirical estimators does not perform well, see Figure 5. We did not see better adherence of the Type I error rates to the nominal levels with transformations. Using the robust SEs with 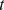-quantiles with 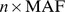 degrees of freedom provided the best adherence of the Type I error rates to their nominal value.

### Power

**Figure 6:**
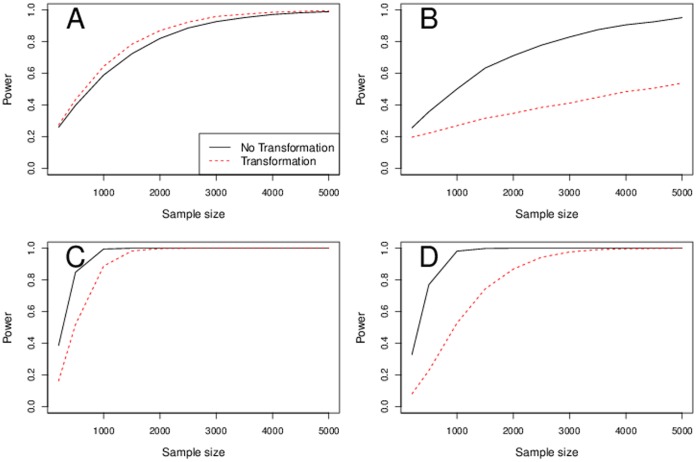
Power in typical scenarios. Weibull distributed errors and MAF 0.3, model-based SEs and normal quantiles. Panel A shows no heteroscedastic data. Panel B shows heteroscedastic data. Panels C and D show power in a situation when a genotype has a large effect in a population subset. Panel C shows no heteroscedastic data. Panel D shows heteroscedastic data.

In non-heteroscedastic data we saw slight power increase with transformations in distributions with heavy tails. On the other hand, in data with heteroscedasticity we saw a large decrease of power with transformations. This is common across MAF, sample size, and all three approaches of combining SEs and quantiles. See Figure 6, panels A and B.

In situations where a genotype has a large effect in a population subset, transformations do not increase power, see Figure 6, panel C for data with no heteroscedasticity and panel D for data with heteroscedasticity. On the contrary, transformations attenuate the signal.

### Data

**Figure 7:**
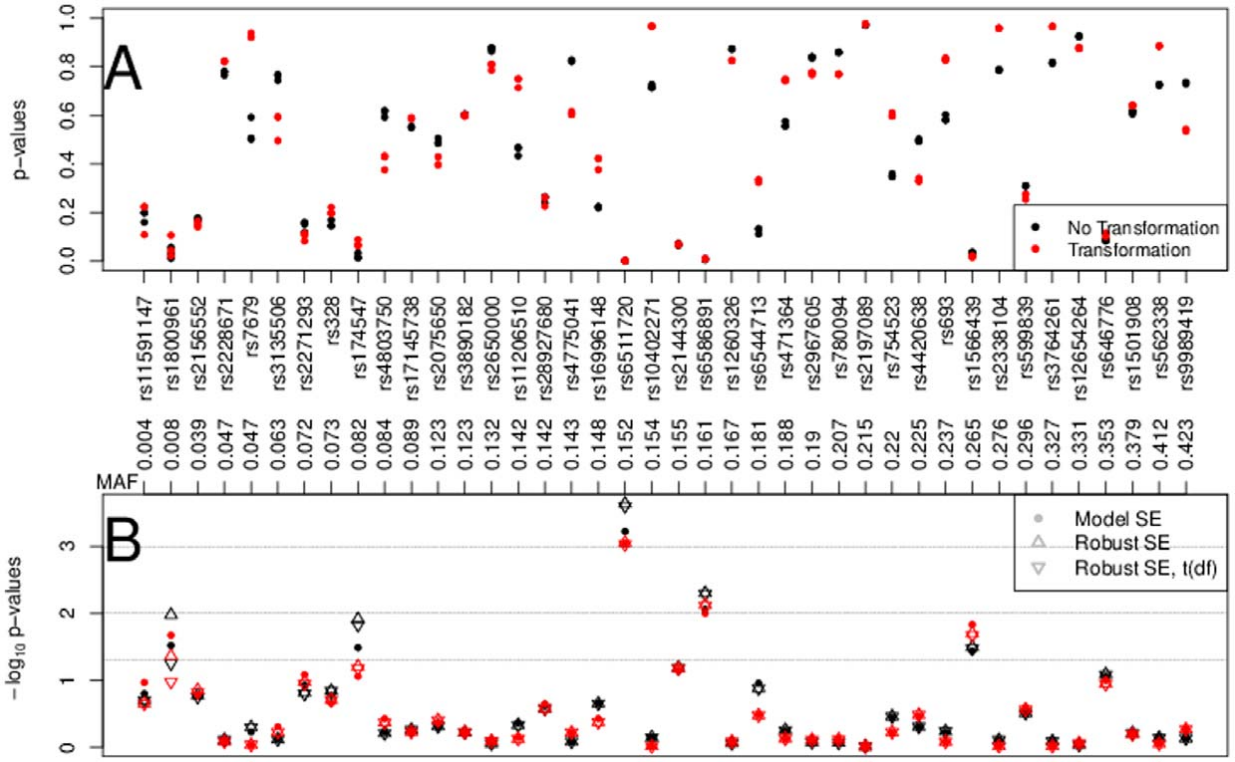
P-values in CHS for the association of LDL-C and 40 SNPs in 799 individuals of African American ancestry. Panel A shows p-values. Panel B shows p-values on the −log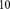 scale to emphasize small values, distinguishing among the three approaches of p-value computation. SNPs are ordered by MAF.

In Figure 7 we present the p-values obtained in the CHS analysis. Panel A, not distinguishing among the three statistical approaches of p-value computation based on SEs and quantiles, illustrates the LDL-C transformation effect on the p-values as compared to the p-values when no transformation was used. For most SNPs the p-values are different. For few SNPs the transformation has little impact, e.g., SNP rs3890182. Comparing the three approaches, with lower MAF the p-values tend to spread out more. For higher MAF the normal and t-quantiles with 799
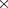
MAF approximate effective degrees of freedom are similar. To study in detail the low p-values of interest, see panel B. For several SNPs, e.g., rs1800961 and rs174547, we see that the LDL-C transformation and the method for computing p-values may result in different conclusions about the association of LDL-C and the SNP.

## Discussion

Linear regression does not require normal distribution of outcome or residuals for sufficiently large samples where Central Limit Theorem applies. In genetic association models sufficiently large is often under 500, depending on the targeted significance level. Sample sizes of studies involved with genetic analyses are mostly substantially larger. Transformations improving outcome normality do not typically improve inference validity. On the contrary, using them may provide invalid inference, specially with heteroscedastic data. Unintuitivelly, under heteroscedasticity transformations proved even less suitable for heavy tailed errors than for normal errors. With this paper we would like to discourage researchers to use phenotype non-normality as an argument to transform it. Model choice should be driven in the first place by biological plausibility and relevance to the scientific question, not by presumed statistical modeling needs.

The extend of the heteroscedasticity problem depends on the skewness of the genotype. Simulations suggest that when ignoring the combination of heteroscedasticity and rare variants statistical inference results may be rather misleading. Specifically, we saw largely inflated Type I error rate, resulting in spurious detection of associations. Robust standard errors must be carefully considered to construct a test statistic. With small sample size t-quantiles with appropriate degrees of freedom might be preferred to normal quantiles, improving adherence of the Type I error rate to the nominal level. We encourage researchers to report in their papers' method sections the choice of standard errors method and the test statistic distribution. The excess of false positive findings that we saw in the simulations due to rare genotype and heteroscedasticity may be one of the many reasons why genetic association findings are difficult to replicate.

## Methods

For each individual 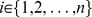 we generate allele 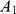 and 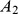 as independent binary variables with probability MAF. We define genotype with an additive model 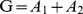, taking on values 0, 1, or 2. We consider MAF 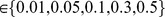. The skewness of genotype G with such minor allele frequencies is about 7, 3, 2, 0.6 and 0, respectively. We generate covariates Age as Poisson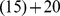 and a Male indicator as binary with probability 0.5. We generate a positive continuous phenotype 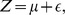 where 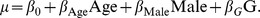


First, we generate data with no heteroscedasticity, generating the errors 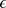 as 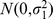, zero-centered 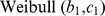 and zero-centered 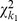. Second, we generate data with heteroscedasticity. We consider the error distribution to be a mixture of normal or centralized Weibull or centralized 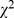 distributions based on the genotype G
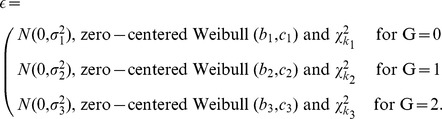



**Figure 8:**
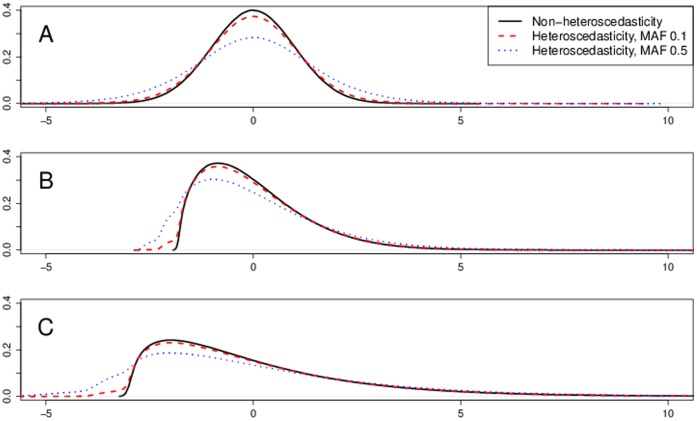
Density of errors 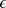 used in simulations. Panel A shows normal errors, panel B Weibull errors and panel C 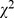 errors.

For normal distribution we set 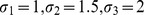. For Weibull distribution we set 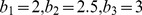 and 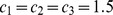, and for 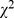 distribution 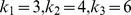. See Figure 8 for density of the generated errors.

We set 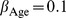 and 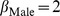. The intercept 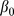 is in Weibull case 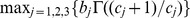, in 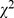 case 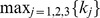, to insure positive values of phenotype. We set 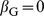 to study Type I error rates. We set 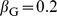 to study power. Additionally, we consider a scenario where a genotype has a fairly large effect in a population subset, perhaps because of an unmeasured environmental exposure 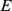. We generate 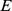 as binary with 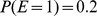 and set 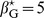.

For large number of simulations under the null hypothesis the empirical 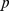-values for valid tests should is uniform distributed on 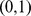. As a summary display of comparison of distribution of the 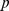-values and a uniform 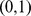 variable we present quantile-quantile plots. We use the 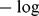 scale to emphasize the area of interest of small p-values and simplify the plots' readability with base 10. We highlight the significance level of 0.05, 0.01, and 0.001. We consider sample size 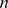 = 200, 500, 1000, 2000 and 5000. We systematically summarize type I error rates in tables across MAF and sample size for significance levels 0.05 and 0.001. Our results are based on 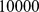 simulations.

Because the quantiles of normal distribution and 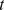-distribution with larger number of degrees of freedom are very close, we do not plot a separate curve for 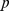-values based on model-based SEs and t-quantiles as it is very similar to the one based on model-based SEs and normal quantiles (the smallest sample size in our setting is 200, resulting in 200-4 = 196 degrees of freedom). This is not so with the robust SEs where the approximate number of degrees of freedom can be very low (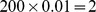), resulting in the t-quantiles being much larger than corresponding normal quantiles, especially for lower significance level. As the log transformation and the INT provided similar results we show the log transformation results only. Similarly, as using the Weibull errors and the 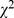 errors provided similar findings, we show the Weibull errors findings only. Simulations were performed in R [22].

We demonstrate the methods using Causal Variants Across Life Course (CALiCo) with data from Cardiovascular Health Study (CHS) [23]. Specifically, we study the association between low-density lipoprotein cholesterol (LDL-C) in 799 individuals of African American ancestry and 40 SNPs as described previously [24]. The linear regression models are minimally adjusted for age and gender. As the log transformation and the INT provided similar results we show the log transformation results only.
